# Curcumin Nanoformulations with Metal Oxide Nanomaterials for Biomedical Applications

**DOI:** 10.3390/nano11020460

**Published:** 2021-02-11

**Authors:** Anteneh Marelign Beyene, Mohammad Moniruzzaman, Adhimoolam Karthikeyan, Taesun Min

**Affiliations:** 1 Department of Animal Biotechnology, Jeju International Animal Research Center (JIA) & Sustainable Agriculture Research Institute (SARI), Jeju National University, Jeju 63243, Korea; antethesecond@gmail.com (A.M.B.); monir1983@jejunu.ac.kr (M.M.); 2 School of Chemical and Bioengineering, Addis Ababa Institute of Technology (AAiT), King George VI St., Addis Ababa 1000, Ethiopia; 3 Subtropical Horticulture Research Institute, Jeju National University, Jeju 63243, Korea; karthik2373@gmail.com

**Keywords:** curcumin, metal oxide nanoparticles, nanoformulations, biomedical applications

## Abstract

In the past few decades, curcumin, a natural polyphenolic phytochemical, has been studied for treating a wide variety of diseases. It has shown promising results as a potential curative agent for a variety of diseases. However, its inherent limitations, such as poor aqueous solubility, poor absorbability, fast metabolic rate, and quick elimination from the body, have limited its application beyond preclinical studies. A huge number of studies have been made to address the issues of curcumin and to maximally utilize its potentials. Many review articles have tried to assess and summarize different nanocarriers, especially organic nanocarriers, for nanoformulations with curcumin. Nevertheless, few exclusive reviews on the progress in nanoformulation of curcumin with inorganic nanomaterials have been made. In this review, we present an exclusive summary of the progress in nanoformulation of curcumin with metal oxide nanoparticles. The beneficial feature of the metal oxide nanoparticles used in the curcumin nanoformulation, the different approaches followed in formulating curcumin with the metal oxides, and the corresponding results, protective effect of curcumin from different metal oxide caused toxicities, and concluding remarks are presented in the review.

## 1. Introduction

Turmeric (*Curcuma longa*) is a well-known spice and food colorant belonging to the ginger (Zingiberaceae) family and *Curcuma* genus [1]. It is one of the over 100 species belonging to the *Curcuma* genus [2]. The ground powder of turmeric has long been used as a multifunctional drug in traditional herbal medicines of Southeast Asian countries and China [3,4]. It has been used to treat a wide variety of diseases such as skin disorders, pulmonary and gastrointestinal ailments, as pain relief, wound healing, liver disorders, infectious diseases, abdominal disorders, and a variety of other ailments [5]. In the past few decades, researchers have started to look at turmeric and to explore its potential in modern medicines. Despite the fact that some research argues the whole turmeric exhibits activities superior to that of curcumin alone [6], the bioactive extract of turmeric, curcumin, has been the subject of interest in modern medicine [3].

Curcumin is a multifunctional polyphenolic phytochemical extract of turmeric (*Curcuma longa*). It is most commonly available in the form of a mixture with two other curcuminoids, desmethoxycurcumin (~5%) and bis-desmethoxycurcumin (~18%), in which curcumin (~77%) is the major component [1,5,6,7]. The international standard name of curcumin is 1,7-bis–(4-hydroxy-3-methoxyphenyl)-1, 6-heptadiene-3, 5-dione [1,2]. Several preclinical and clinical [2,5] studies demonstrated that curcumin has antimicrobial, anti-inflammatory, anticancer, antioxidant, and other therapeutic benefits [8]. Exhaustive researches have been made on the application of curcumin for a variety of diseases and on the curing mechanism of curcumin [1]. Despite its therapeutic potential, it is poor aqueous solubility, poor absorbability, fast metabolic rate, high rate of excretion [5,8] and the consequent poor bioavailability of pure curcumin, both in vitro and in vivo, has limited its therapeutic potential. The poor bioavailability and stability of curcumin with its relatively higher microbial inhibitory concentration, compared to other antimicrobial agents, makes it difficult to achieve inhibitory action in vivo [9].

Apart from the limitations of pure curcumin as a therapeutic agent, some researchers have observed the dose-dependent toxicity of curcumin, also raising concerns over the safety of the chemical [7]. According to some reports, higher concentrations of curcumin can cause DNA damage and chromosomal alteration, ulcer, and hypoplasia [10]. Other reports also mentioned iron chelation and consequent causes of overt iron deficiency in the case of subclinical iron-deficient mice [1,6]. Some researchers argue that the relatively low incidence of gastrointestinal cancer at higher consumption of turmeric—up to 1500 mg (~50 mg of curcumin) per person per day in Nepal and 2000–2500 mg (~100 mg of curcumin) in India—is due to the fact that these consumptions are much lower than the dose administered in clinical trials [5,6].

Increasing the bioavailability and stability of curcumin have been the focus of many researchers in recent years. Advances have been made in the past few decades in formulating curcumin with nanocarriers to overcome its limitations. Several reports have demonstrated various nanoformulations of curcumin and the consequent improvements in its aqueous solubility, absorbability, stability, bioavailability, and overall therapeutic potential [11]. The most common approaches to improve the limitations of curcumin have been encapsulating [2] or incorporating (binding) [8] curcumin with other nontoxic, biocompatible materials. Polymeric nanoparticles, micelles, hydrogels, nanoemulsions, liposomes, solid lipid nanoparticles, polymeric nanostructures, and inorganic nanomaterials are some of the widely studied carries. Nanostructures have shown the capabilities of enhancing the therapeutic activities of the drugs by prolonging the half-life of the drug, increasing the solubility of hydrophobic drugs, reducing potential immunogenicity, and releasing drugs in a sustained and stimulant triggered manner. They can offer the possibility to control the rate and site at which the drug may be released [12].

As drug carriers, inorganic nanomaterials, such as metallic nanoparticles, carbon nanotubes, graphene, mineral, and metal oxides, have several advantages over their organic counterparts. Compared to organic drug carriers, inorganic carriers possess better stability, high surface area and porosity, better drug-loading capability, better bioavailability, lower toxic side effects, controllable drug-release capability, tolerance towards most organic solvents, and better functionality [1,2,13,14,15,16,17,18]. Particularly, some metal oxides like TiO_2_, ZnO, Fe_3_O_4_, CeO_2_, and CuO have demonstrated relatively high stability and less toxicity. They can be synthesized through simple synthesis approaches to a desired shape and morphology. They do not show swelling variation, can be incorporated into both hydrophilic and hydrophobic systems, and can easily be functionalized by various molecules due to their negative surface charges [19]. They are relatively low-cost materials and have the potential to complement the therapeutic effect of curcumin as some of them show therapeutic effects themselves. They have been applied in diagnostics, for imaging of different molecular markers, as a photosensitizer in photodynamic therapy, and in targeted delivery of drugs. Metal oxides can increase the stability and bioavailability of curcumin by protecting the encapsulated or bounded curcumin from hydrolysis, phagocytosis and by increasing the aqueous interaction and blood circulation of curcumin [17]. Some studies reported that composite curcumin–metal oxide therapeutic agents demonstrate a better therapeutic performance than the constituents. Usually, the composite drug show additive therapeutic performance [20].

In this review, we first discuss the biomedical related features of metal oxides with which curcumin nanoformulations have been reported. In the next section, the different approaches to formulate curcumin with metal oxide nanoparticles (NPs) with their corresponding outcomes have been summarized. The ameliorative effects of curcumin on metal oxide-caused toxicities have also been discussed. In the final section, the research gaps and future directions have been proposed in the form of concluding remarks.

## 2. Features of Metal Oxides Related to Biomedical Applications

**Figure 1:**
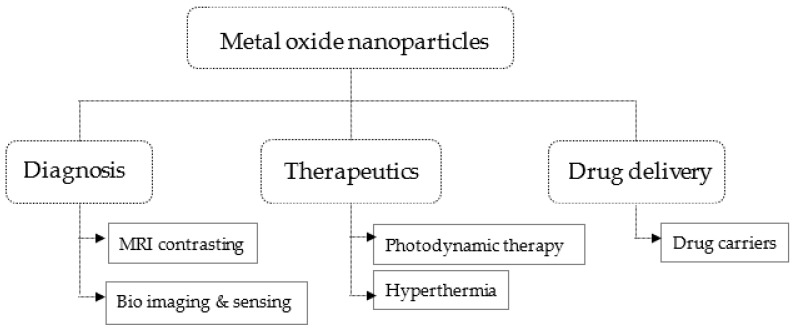
Biomedical applications areas of metal oxides.

Metal oxides reported as a drug carrier for curcumin include TiO_2_, CuO, ZnO, Fe_3_O_4_, and CeO_2_. These metal oxides are observed to have relatively less harm for biological systems [19,21,22] and thus have been applied as a carrier for curcumin. They possess several beneficial features for biomedical applications besides their relatively less toxicity to normal biological cells. The biomedical applications of metal oxides are summarized in Figure 1.

### 2.1. Titanium Dioxides Nanoparticles

TiO_2_ and ZnO NPs are large bandgap semiconductors having bandgap energy of ~3.2 and ~3.3 eV, respectively. Upon UV absorption, excited electron and hole pairs will be generated in these semiconductors. These electron–hole pairs react with the atmospheric oxygen and moisture to generated reactive oxygen species (ROS) [23,24]. The highly reactive ROS decomposes any organic matter, including the cell membranes of biological organisms leading to cell leakage and death. TiO_2_ is the most preferred photocatalyst for industrial-scale photocatalyst in terms of photoactivity efficiency, stability, and the coast [25]. The anatase phase of TiO_2_ has an indirect bandgap thus has a relatively longer recombination time consequently is a better photocatalyst than the direct bandgaps of rutile and brookite. Chemical stability, high opacity, low toxicity, and bio friendliness are the other notable properties of TiO_2_. Apart from its conducive physicochemical property and biocompatibility, it is an abundant and low-cost material. Biomedical application of TiO_2_ encompasses its use as a therapeutic agent in photodynamic therapeutics [26], as a fluorescent probe and contrasting agents in diagnosis [27], and as biocompatible and efficient drug carriers for both hydrophobic and hydrophilic therapeutic drugs [28]. Although the toxicity of NPs dependent on several factors, some studies revealed that TiO_2_ NPs are less toxic compared to other metal oxides like ZnO and CuO NPs of the same particle size at the same concentration [29,30,31]. TiO_2_ particles in the micrometer range have shown more toxicity towards human lung epithelial cells (A549) than those in the nanometer range in a size-dependent toxicity study [32].

### 2.2. Zinc Oxide Nanoparticles

Zinc oxide has excellent ultraviolet absorbability and visible light transparency [24]. It has been reported that ZnO NPs with particle sizes greater than 100 nm is considered to be relatively biocompatible [24]. It is labeled as generally reconsider safe (GRAS) by the United States Food and Drug Administration (FDA) [21]. It does not interact with major pharmaceutical active elements available, which makes it a good candidate for drug delivery. ZnO crystals have a large number of valance band holes and conduction band electrons even without being irradiated with UV light because of defects [24]. It exhibits excellent antibacterial, anticancer, anti-inflammation, and wound healing properties [33]. Its biomedical application is mainly dependent on its ability to trigger ROS generation, release Zn^+2^ ion, and induces cell apoptosis. However, in the particle size range less than 100 nm, it has a higher degree of toxicity to normal cells compared to other metal oxides [34]. The higher level of dissolution and consequent Zn^+2^ ion formation has been associated with a relatively higher degree of toxicity [35]. ZnO shows excellent luminescence properties and thus is one of the main candidates for bioimaging [33].

### 2.3. Iron Oxide Nanoparticles

Superparamagnetic iron oxide NPs (SPIONs) are receiving attention in the biomedical fields, such as contrasting agents in MRI imaging, cell separation and detections, drug delivery, and hyperthermia [36,37]. Their intrinsic magnetic properties can be utilized for external magnetic field derived targeted drug delivery and for inducing local hyperthermia. A study observed Fe_2_O_3_ and Fe_3_O_4_ particles had shown size-independent (i.e., no difference between a micrometer and nanometer-size particles), low toxicity on A549 cells compared to those of TiO_2_ and CuO particles [32]. Surface coating is necessary while using SPIONs to avoid their high agglomeration tendency and biofouling of the NPs in blood plasma [17]. They are also less toxic than other magnetic materials [38], biocompatible, and biodegradable, and can be cleared from the body via iron metabolisms pathways [18].

### 2.4. Cerium Oxide (Ceria) Nanoparticles

CeO_2_ NPs possess a large number of oxygen vacancy defects at the surface of ceria NPs due to the interconversion of oxidation states. CeO_2_ coexists in both Ce^+3^ and Ce^+4^ valance states. These unique redox cyclic potential from Ce^+3^ to Ce^+4^ has attracted interest in the biomedical research fields. It is a convenient material for treating ROS mediated disorders such as neurogenerative disorder, retinal disorder, cancer, and inflammation, and others [39,40]. It is observed that CeO_2_ acts as a catalyst that mimics the features of antioxidant enzymes such as superoxide dismutase (SOD), catalase, and oxidase, in terms of scavenging ROS and free radicals [31,41,42]. Nanoceria has been reported to cause selective toxicity towards human lung cancer cells and prostate cancer cells but has shown no toxicity to normal cells (L929). However, higher doses, i.e., 1000 mg/kg per body weight, of ceria NPs were reported to cause DNA damage in liver and peripheral blood leukocytes [43].

### 2.5. Copper Oxide Nanoparticles

Copper oxides are a narrow bandgap (~2 eV) p-type semiconductor. CuO NPs possess properties like good electrochemical activities, proper redox potential and excellent stability in solutions. CuO NPs have been employed in sensing and targeting both in vitro and in vivo. CuO NPs have been recognized as antimicrobial material by the US environmental protection agency (EPA). They have demonstrated their antimicrobial effect through their antitumor, antibacterial, and antifungal properties [44,45]. CuO also has demonstrated antioxidant properties. According to reports, the toxicity of CuO NPs is not selective, and it is also toxic to normal cells [46,47]. A study obtained the highest toxicity in the case of CuO treatment of A549 cells compared to the results of ZnO, TiO_2_, Fe_2_O_3_, Fe_3_O_4_ NPs treatments at similar concentrations [48]. Another study observed the nano-sized particles of CuO to be more toxic on A549 cells than micro-sized CuO particles [32]. The toxicity is mainly due to the production of ROS, which can cause oxidative stress and consequent cell damage. Dissolved metal ions are also associated with the toxicities of CuO [49]. Intracellular ROS generation by CuO NPs initiated by the catalysis of free radicals in the mitochondria [50].

## 3. Metal Oxides as Curcumin Carriers

### 3.1. Metal Oxide–curcumin Direct Combination

**Figure 2:**
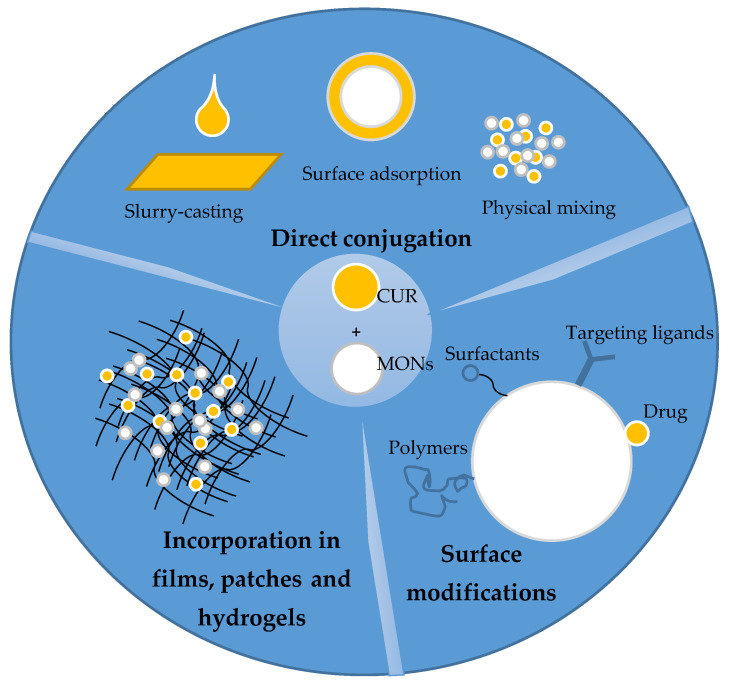
Schematic representation of the different approaches in curcumin–metal oxide nanoformulations.

Several approaches have been followed to load curcumin on metal oxides vehicles and effectively deliver them (Figure 2). Direct conjugation of curcumin with metal oxides by adsorption on the metal oxides surfaces, simple mixing of curcumin with metal oxide nanoparticles, and curcumin–metal oxides slurry-casting have been reported (Table 1). Sherin et al. [51] reported the effective adsorption of curcumin on TiO_2_ nanoparticles surface and were able to obtain stable curcumin–TiO_2_ nanocomposite having a size of ~29 nm and zeta potential of -53.7 mV. The drug loading efficiency of the TiO_2_ NPs was 48%. Curcumin has also been loaded directly on the surface TiO_2_ nanotube arrays, which were used to modify the surface of Ti6Al4V implant via electrochemical anodization, using the drop-casting method. The design was intended to address issues of infection around titanium orthopedic implants. The casting of curcumin on the TiO_2_ nanotube array has given the implant hydrophobic characteristics, which enables it to limit initial bacterial adhesion. The curcumin casted TiO_2_ array has further shown antibacterial effect by causing 43% and 38% reduction of *Escherichia coli* (*E. coli)* and *Staphylococcus aureus* (*S. aureus)* population respectively [52]. Inhibition of HeLa cancer cells has also been reported by directly-conjugated curcumin–TiO_2_ nanocomposite [53]. Oh et al. [54] observed at subinhibitory concentration, curcumin adsorbed on the surface of Ti and TiO_2_ implants rather enhance bacterial adhesion.

Perera et al. [20] synthesized ZnO NPs with different morphologies and performed surface adsorption of curcumin on the nanoparticles. According to their study, the loading efficiency of curcumin on ZnO NPs indicated that long-petal and javelin morphologies possess higher loading. However, the antimicrobial, anticancer, and cytotoxicity of the nanocomposites have not shown a particular trend with the curcumin-loading amount, indicating other nanoparticle characteristics like morphology and surface properties also have an impact on the therapeutic performances besides the amount of loading. Core–shell curcumin–ZnO composite has been synthesized using simple precipitation of curcumin under ultrasonication. The synthesized core–shell structure, with 45 nm ZnO core and 12 nm curcumin shell, has shown complete water dispersibility. The core–shell curcumin–ZnO nanocomposite demonstrated a better antibacterial performance against *Staphylococcus pneumoniae (S. pneumoniae)* and *E. coli* than commercial antibiotic amoxicillin [55]. A study reported the ameliorative effect of co-administered curcumin and ZnO NPs to the hepatotoxicity caused by aflatoxin B1 (AFB1) on AFB1-fed rabbits. ZnO and curcumin demonstrated hepatoprotective effect through scavenging free radicals and by enhancing the activities of antioxidants superoxide dismutase (SOD), catalase (CAT), and glutathione peroxidase (GSH-Px) [56].

Completely water-dispersible curcumin–copper oxide (CuO) core–shell structures have been developed by direct precipitation of curcumin under ultrasonication. The developed core–shell structure has shown a better water solubility than nano curcumin or CuO NPs alone. The antibacterial performances of the core–shell structure against *E. coli*, *S. aureus*, *Shigella dysenteriae (S. dysenteriae),* and *S. pneumoniae* were better than that of nano curcumin or commercial amoxicillin. The authors observed the antibacterial effect of the core–shell structure to have an inverse relation to the amount of curcumin shell, which they attributed to the reverse electron transferase reaction caused by curcumin via supper oxides. On the other hand, the core–shell structure has shown less toxicity than CuO NPs on African green monkey kidney cells (Vero) [57]. A curcumin–CuO complex made by grinding together commercial curcumin and CuO has been reported for biomedical applications. The developed nanocomplex has been tested for its anti-diabetic effect in vivo on streptozotocin (STZ) induced diabetic mice. Curcumin–CuO treatment resulted in a significant decrease in malonaldehyde (MDA) levels, increase in GSH and SOD levels, increase in insulin concentration, and upregulated mRNA-expression levels of insulin gene (IR-A and IR2), compared to the results of pure curcumin treatment [58]. Bhandari et al. [59] reported a direct coating of Fe_3_O_4_ NPs with curcumin and tested the antioxidant property of the composite. The resulting curcumin-coated iron oxide demonstrated lower toxicity than uncoated Fe_3_O_4_ or pure curcumin to human umbilical vein endothelial cells (HUVEC). The composite also demonstrated a protective effect against polychlorinated biphenyl 126 (PCB126) exposed HUVEC. The biocompatibility and the protective effect of nanocomposite against inflammatory agent PCB have been attributed to the antioxidative property of curcumin.

### 3.2. Surface-Modified Metal Oxides as Curcumin Carriers

Functionalization of metal oxide NPs prior to curcumin-loading has been reported by several researchers. Surfactants, targeting ligands and antibodies, cellulose-based polymers, chitosan and its derivatives, dextrin and derivatives are among the most-reported materials for the surface modifications of metal oxides. Functionalization of metal oxide nanoparticle surfaces is usually made to prevent agglomeration and opsonization, prolonged circulation time in body fluids, enhanced target specificity, and prolonged ROS life span in the case of photocatalytic metal oxides like TiO_2_ and ZnO. Table 2 summarizes the different surface modifications and the corresponding benefits in metal oxide–curcumin nanoformulations.

#### 3.2.1. Targeting Ligands Modification

Compared to normal cells, cancer cells excessively express several normal proteins on their surface. These overexpressed proteins can be used as targets in a targeted delivery system. Such ligand-mediated targeting is known as active targeting. Peptides, proteins, nucleic acids, carbohydrates, antibodies and small molecules like folates are commonly used as targeting ligands [60].

Folic-acid-conjugated, polyethylene glycol-functionalized TiO_2_ (FA/PEG/TiO_2_) has been reported as a carrier for curcumin/salvianolic acid B codelivery. The codelivery of salvianolic acid B was intended to impart a cardioprotective effect against TiO_2_ NPs caused injury. The viability of cardiac myoblast (H9c2) with salvianolic B was higher than that with only TiO_2_ NPs, indicating the protective effect of Sal B on cardiac cells against TiO_2_ induced injury. The viability and cytotoxicity of both MCF7 and MDA-MB-231 revealed that the nanodrug complex with FA targeting ligand showed better performance than the untargeted drug complex. In vitro cellular uptake was higher with FA than without FA. In vivo biodistribution and antitumor activity of the drug in MDA-MB-231 tumor-bearing BALA/c, nude mice revealed higher drug concentration at the tumor site and better antitumor activity in terms of tumor growth reduction in the case of FA-conjugated composite drugs [61]. Similarly, Hermat et al. [62] reported codelivery of curcumin and paclitaxel by active folic acid (FA) and passive magnetic NPs targeting. The magnetic NPs were functionalized by oleic acid surfactants and further conjugated with PF127 and PF127FA. Paclitaxel (PXT) and curcumin (CUR) loaded nanocomposite demonstrated an efficient encapsulation of 37.9% PTX and 56.5% CUR. Cellular uptake and cytotoxicity of the designed drug against MCF-7 have been promoted by the active FA and passive magnetic field targeting. A relatively lower IC_50_ value has been observed in the case of PTX–cur-OAMNPPF127FA under an external magnetic field. Another study reported Folic acid decorated, human serum albumin and citric acid-functionalized, Fe_3_O_4_ for the codelivery of 5-fluorouracil and curcumin. The designed composite demonstrated good colloidal stability with a zeta potential of −49 mV. The composite nanoparticle demonstrated a good magnetic property with magnetic saturation of 33.59 emu/g without hysteresis. The cytotoxicity assay revealed that FA-conjugated nanocomposite leads to lower viability of MCF-7 cells. In the presence of a magnetic field, the cytotoxicity of the composite drug was further enhanced. Cellular uptake increased 1.32 times due to FA and 1.9 times due to magnetic fields targeting after 1h incubation [63]. Saikia et al. [64] studied folic-acid-conjugated, aminated starch/ZnO-coated Fe_3_O_4_ for curcumin delivery. They obtained enhanced cellular uptake of the composite by HepG2 cell due to folic acid targeting. The zeta potential value of the composite drug was 42.9 mV indicating higher colloidal stability of the design. The composite drug demonstrated less toxicity to human lymphocytes while showing higher inhibition against MCF-7 and HepG2 cancer cells. Therapeutic performance enhancement through folic acid conjugation has been studied using curcumin-loaded, folic-acid-conjugated, Fe_3_O_4_ NPs incorporated methoxy-PEGylated poly (amidoamine) (PAMAM) generation 3 dendrimer. The cell lethality was 65% for the nanocomposite treated KB cells (with higher FA receptors), while it was only 38% for the case of MCF-7 cells (with lower FA receptors) [12].

Sherin et al. [65] reported an MCP-1 antibody-conjugated curcumin–TiO_2_ (CTNPs) nanocomposite for an early diagnosis and treatment of atherosclerosis. They dispersed presynthesized curcumin–TiO_2_ nanocomposite in MCP-1 antibody solution under stirring to make the antibody conjugation. The MCP-1-conjugated CTNPs resulted in a good contrast image during MRI scanning at the aortic region of atherosclerotic rats, while the image was of low contrast in the case of normal mice. CTNPs without antibody conjugation resulted in an image with a nuclear pattern of contrast, which was associated with the low concentration of CTNPs at the tumor site. In vitro and in vivo toxicity analysis of the designed nanocomposite revealed that their designed contrasting agent was nontoxic, and the authors attributed the nontoxicity to the anti-toxic effect of curcumin.

#### 3.2.2. Surfactant and Other Organic and Inorganic Compounds Modification

β-Cyclodextrin (CD) is one of the polymeric materials that was reported for surface modification of metal oxide nanoparticles. Surface modification with β-CD has been reported to bring about stability of nanoparticles, higher drug loading, controlled drug release, and reduced protein corona adsorption on the nanoparticles. β-CD has the potential to form a reversible inclusion complex, which enables it to encapsulate the hydrophobic drug and release it in a sustained manner. Meng Zhang et al. [66] reported curcumin-loaded, dopamine and β-CD-functionalized, TiO_2_-based implant for preventing postoperative recurrence caused by incomplete removal of the tumor during surgical treatments. In their design, the polydopamine coating was intended to create a robust anchorage of β-CD, which serves as a curcumin reservoir. β-CD coating enhanced the wettability and thus bioavailability of curcumin besides providing efficient drug loading and release. Human osteosarcoma (MG63) cells cultured on the designed drugs with different curcumin-loading demonstrated pronounced membrane shrinkage, round cell morphology without filopodia and lamellipodia, and low cell viability. However, no adverse effect has been observed on embryonic osteoblasts (MC3T3-E1) cells cultured in the same condition. The results demonstrated the selective antitumor effect of the designed nanocomposite. In vivo analysis also has demonstrated a significant decrement in tumor growth of osteocarcinoma xenografted nude mice upon treatment with the designed nanocomposite. Another study reported hydroxyapatite (HAPA)-coated, β-CD-functionalized superparamagnetic NPs (SPIONs) for the codelivery of doxorubicin (DOX) and curcumin. β-CD functionalization significantly reduced the amount of protein corona adsorbed on the composite compared to that adsorbed on nanocomposite without β-CD functionalization. Significant cellular uptake of the nanocomposite by MCF-7 cells has been observed, which the authors attributed to the protein corona impairment by β-CD. The nanocomposite demonstrated hemocompatibility with only 0.1% hemolysis at 2 mg/mL concentration. It also has demonstrated higher toxicity on MCF-7 cells than the drugs (DOX and CUR) mixture alone. In vivo study on tumor-bearing BALB/c mice revealed the lowest percent relative tumor volume (% RTV) for DOX-CUR/NCs treatment with magnetic field compared to that of free DOX. The P-gp expression is suppressed in the case of curcumin incorporated composite drug, and the magnetic field further enhanced the suppression [67].

A monodispersed iron oxide NPs have been synthesized through coprecipitation in the presence of β-CD and subsequent pluronic F68 polymer coating. The amphiphilic β-CD/F68 coating facilitates interaction between the hydrophilic Fe_3_O_4_ and the hydrophobic payload, curcumin. The designed nanoformulation resulted in a less aggregated nanocomposite with a hydrodynamic size of 123 nm, negative zeta potential, and a smaller polydispersity index (PI 0.172). Slow and sustained release of curcumin was possible due to MNPs (47% released in 5days). Drug uptake by the MDA-MB-231 cells has shown receptor-mediated targeting. Treatment of MDA-MB-231 cells with the composite drug demonstrated morphology change such as membrane shrinkage and vacuoles formation and reduced cell viability. The nanocomposite drug has shown effective prevention of serum protein adsorption and colony formation [68]. In another study, functionalization of ZnO NPs with PEG and β-CD for high curcumin- loading has been reported. They were able to obtain higher drug encapsulation efficiency, and pH-dependent sustained drug release because of the β-CD coating. The antibacterial and anticancer properties of the curcumin-loaded, PEG-β-CD-functionalized ZnO NPs were more effective than pure curcumin. β-CD-functionalized nanocomposite has also shown better photoluminescence property compared to only PEG-functionalized composite [69]. Yallapu et al. [70] developed curcumin-loaded β-CD and pluronic F127 stabilized iron oxide for hyperthermia, magnetic resonance imaging, and drug delivery applications. The formulation resulted in a water-dispersible nanocomposite with efficient drug encapsulation and sustained release. They obtained superior hyperthermia effect under an alternating magnetic field, improved MRI contrasting during MRI scanning of ovarian cancer cells (A2780PC), and inhibition effect against A2780PC, MDA-MB-231, and prostate cancer (PC-3). In a separate report, the same group of researchers used the same materials for pancreatic cancer treatment. In vitro cellular uptake of the NPs was tested on HPAF-II and Panc-1 cancer cell lines and exhibited efficient uptake on dose-dependent manner. The nanocomposite also demonstrated effective inhibition of cancer cell growth via hindering proliferation and colony formation. In vivo tumor growth reduction by the nanocomposite also has been observed on HPAF-II xenograft mice. The nanoformulation increases serum bioavailability of curcumin 2.5 times than that of pure curcumin [71].

Chitosan is another common natural biopolymer used for surface modification of inorganic nanoparticles. The cationic nature of chitosan makes it have better interaction with the anionic cell membranes and subsequently leads to its applications as an adsorbent, antibacterial membrane, and drug delivery agent [72]. Pharm et al. [73] reported a core–shell superparamagnetic iron oxide (SPION)—chitosan polymer as a delivery vehicle for curcumin. The core–shell structure demonstrated a controlled drug release profile after initial burst release. They observed that the paramagnetic property of the SPION was present even after the chitosan coating. The curcumin-loaded core–shell structure has shown better cytotoxicity against A549 cells (IC50 11.37 µg/mL) than of free curcumin (IC50 73.03 µg/mL). Upadhyaya et al. [74] have used a water-soluble derivative of chitosan, O-carboxymethyl chitosan (O-CMCS), for surface coating of ZnO in the curcumin/ZnO drug delivery system. They obtained better water solubility of curcumin in the composite form than in pure form. Cur-O-CMCS-ZnO has shown preferential accumulation and cytotoxicity towards MA104 cancer cells compared to normal L929 cells. Venkatasubbu et al. [75] obtained a bactericidal antimicrobial effect when they used chitosan/TiO_2_-curcumin to treat wound infection-causing bacteria (*S. aureus* and *E. coli*) both at lower and higher concentrations, whereas the antibacterial mechanics was bacteriostatic at a lower concentration in the case of curcumin only samples. In another report, hydrophobic (phendione)-modified chitosan-conjugated CuO NPs were used as an efficient curcumin carrier. The hydrophobic modification increases the interaction of the carrier with the hydrophobic drug. The surface modification, along with the high surface adsorption potential of CuO NPs resulted in high drug loading efficiency (96.3%) and sustained drug release. The anticancer performance of the composite against skin cancer cells (M19-MEL), breast cancer cells (MCF-7), and ovarian cancer cells (HeLa) revealed twice lower IC_50_ values than that of free curcumin [76]. Sawant et al. [77] obtained better antimicrobial performance against *Shigella* bacteria using curcumin-loaded, chitosan-coated anatase TiO_2_ NPs compared to curcumin–TiO_2_ mixture without chitosan. In another study, chitin-glucan from bio source has been reported as a reducing and capping agent in ZnO nanoparticle synthesis for curcumin delivery. The resulting curcumin-loaded, chitin-glucan-functionalized ZnO NPs (Cur–ChGC@ZNONPs) have shown a better inhibition effect against *E. coli* and *B. subtilis* growth. The antioxidant effect of curcumin-loaded samples, as evaluated by DPPH and ABTS assay, demonstrated increased scavenging activities as the concentration Cur–ChGC@ZNONPs increases. Both antimicrobial and antioxidant properties of Cur–ChGC@ZNONPs were better than ChGC@ZNONPs [78].

PEGylation of NPs has been reported to reduce opsonization and increase the circulatory retention time of NPs in body fluids [79]. Dhivya et al. [80] reported PEG–PMMA copolymer modified ZnO NPs for curcumin delivery. The designed copolymer encapsulation on ZnO NPs facilitated the efficient incorporation of curcumin via hydrophobic-hydrophobic interaction. They obtained 47% drug loading percentage and 92% loading efficiency. In vitro drug release study revealed that release rate was faster in acidic medium, pH 5.8 than in neutral medium, pH 7.2 (normal cells and tissue environment). They attributed the faster release rate in acidic conditions to the condensation of the hydroxyl group in the copolymer in an acidic environment, which loosened the binding of curcumin to the composite. The IC_50_ value, against AGS cancer cells, of ZnO nanoparticles, curcumin nanoparticles, and the composite drug were 0.05 µg/mL, 0.05 µg/mL, and 0.01 µg/mL, respectively. More pronounced DNA fragmentation has been observed in the case of composite treatment. The nanocomposite demonstrated an enhanced anticancer effect compared to its constituent nanoparticles. PEG-functionalized, gold nanoparticle decorated, Fe_3_O_4_–silica core–shell structure has been reported for magnetoplasmonic diagnostics and therapeutic drug delivery applications. They obtained a stable composite assembly with hydrodynamic size 140 nm and zeta potential of −24.5 mV. MPA-PEG–curcumin assembly demonstrated MRI imaging and anticancer potentials by resulting in a lower T2 value and 53.4% viability in 42 h when applied in HL60 cancer cells. The highest anticancer effect has been observed for PEG-functionalized samples compared to samples without PEG or only magnetic nanoparticle core [81]. A more water-soluble form of curcumin, curcumin diglutaric acid (CG) loaded on PEG–chitosan oligosaccharide-coated SPIONs has been studied for its anticancer effect. The nanoformulation resulted in a stable nanocomposite with a particle size of 130 nm and zeta potential of 30.6 mV. A low level of protein binding has been observed in PEG-functionalized samples compared to other samples without PEG. The cytotoxicity of the composite against human colorectal adenocarcinoma (HT-29) cells was better compared to CG or the functionalized NPs without CG. They observed a further increase in cytotoxicity by applying an external magnetic field [82]. Chen et al. [80] reported PEG-modified CeO_2_@SiO_2_ for codelivery of proanthocyanidin (PAC) and curcumin (cur). PEG functionalization reduced the hydrodynamic size of the composite from 421 to 359 nm. The nanocomposite demonstrated strong antioxidant activity, potent neuroprotective effect against Aβ_1-42_ mediated toxicity in PC-12 cells, and less cytotoxicity towards L02 cells; whereas, it caused an antiproliferative effect against HepG2 and HeLa cells.

A water and alcohol soluble nanoceria–curcumin conjugate has been synthesized by co-vaporization with poly (N-vinyl pyrrolidone), PVP. The conjugation of curcumin with nanoceria significantly increased the aqueous and photostability of curcumin and also led to a two-fold increase in the antioxidative effect of curcumin. PVP functionalization, on the other hand, resulted in an increase in cellular uptake of both free curcumin and ceria-conjugated curcumin [83]. Other surfactants such as oleic acid and citric acid have been used to modify the surface of Fe_3_O_4_ prior to curcumin-loading. The composite drug has shown better anticancer effects by causing lower viability of MDA-MB-231 cells compared to pure curcumin treatment. The composite demonstrated superparamagnetic property with a saturation magnetization of 60–80 emu/g. It also has shown MRI contrasting potential by demonstrating a reduced T2 relaxation time [84].

Curcumin modification with glycidyl trimethyl ammonium chloride (GTMAC) and subsequent conjugation with ZnO NPs has been reported by Pourhajibagher et al. [85] for the incorporation of antibacterial filler in Transbond (TX) orthopedic adhesive. A photoactivated, 7.5 wt % curcumin–ZnO containing adhesive has shown antibacterial growth inhibition for 90 days, and even after 90 days, the bacterial growth rate was lower than that of the control sample as observed by zone of inhibition in disc diffusion analysis. The incorporation of antibacterial fillers has not led to compromise in mechanical strength; shear bond strength (SBS) of the sample was in the acceptable range (6–8 Mpa). The authors attributed the antibacterial effect to the photodynamic therapeutic (PDT) effect of curcumin under visible light irradiations.

A curcumin–ZnO incorporated carboxymethyl cellulose (Cur/ZnO/CMC) has been studied for cancer treatment. CMC has been used to enhance the water solubility of the drug. The composite matrix has shown a loading efficiency of 44% and a pH sensitivity, controlled release profile. The composite has shown aqueous solubility, unlike pure curcumin. The viability of L929 cells was 80% when treated with Cur/ZnO/CMC, whereas that of MA104 cells was 20%, indicating the selective cytotoxicity of the composite to cancer cells [86]. Natural polyphenols-conjugated carboxylated TiO_2_ NPs have been evaluated for their antioxidative effect. Among the conjugate polyphenols—curcumin, quercetin, catechin, and vitamin E—curcumin-conjugated TiO_2_-based composite has shown better antioxidant effect both in DPPH and LPO assays. All phenolic and TiO_2_-based composite demonstrated less cytotoxicity towards intestinal Caco2 cell lines [87]. Carboxy terminated ZnO nanoparticle has been synthesized via coprecipitation of ZnCl_2_ in the presence of 3- mercapto propionic acid for curcumin delivery. The functionalized composite demonstrated a significant increment in aqueous solubility and decrement in IC_50_ against MDA-MB-23 cells compared to that of free curcumin [88].

Other miscellaneous organic and inorganic surface modifications have also been reported in the curcumin-Metal oxides nanoformulations. Nosrati et al. designed core–shell structure of bovine serum albumin and Fe_3_O_4_–curcumin (F@BSA–curcumin) and has obtained pH-dependent drug release and selective tumor cell cytotoxicity (biocompatible to HFF2 cells, while cytotoxic to MCF-7). The core–shell structure has also demonstrated magnetic susceptibility, although the value is reduced due to curcumin and albumin-coating [89]. Better aqueous dispersibility, room temperature magnetic property, and controlled drug release profile have also been observed through APTES coating of curcumin-Fe_3_O_4_ nanocomposite [90]. A higher curcumin-loading percentage (89%) has been obtained by coating carbon dotes on the surface of rutile TiO_2_ nanoparticles. The curcumin-loaded composite demonstrated significant inhibition of HaCaT cells (60.7% at 100 µg/mL concertation), which was comparable to that of the positive control 5-fluorouracil. The composite also caused selective apoptosis on MCF-7 cells with no adverse effect on mouse fibroblast (McCoy) [91]. A curcumin-loaded graphene–ZnO nanoformulation has been reported for inhibition of MRSA biofilm formation. They obtained a more than a five-fold inhibitory effect on *S. aureus* in the case of curcumin–ZnO incorporated graphene composite compared to independent curcumin or graphene–ZnO [92]. Sudakaran et al. studied the anticancer effect of MgO, curcumin and β-cyclodextrin, aloe vera (AV), incorporated poly (l-lactic acid-o-Ɛ-caprolactam) (PLACL) composite nanofiber against MCF-7 cells. The incorporation of MgO NPs gave rigidity to the nanofibers. PLACL/AV/MgO/CUR nanofiber composite has shown a better anticancer effect by reducing the proliferation of MCF-7 cells by 65.9% compared to PLACL/AV/MgO [93].

### 3.3. Curcumin-Metal Oxide Incorporated Films and Patches

Incorporation of metal oxides together with curcumin in wound dressing patch and packaging films have been reported for antimicrobial and other functional properties enhancement of the corresponding materials. Direct mixing of curcumin and metal oxides in the polymer solutions and subsequent casting were the common approaches followed by the studies to prepare these therapeutic films and patches. The incorporation of curcumin–metal-oxide conjugates in different films, patches, and hydrogels are summarized in Table 3.

Salarbashi et al. [77] reported curcumin and TiO_2_ incorporated soluble soybean polysaccharides for smart food packaging films. They followed the solution-casting method to synthesize the composite film. The composite film containing 15% TiO_2_ and 0.4% curcumin demonstrated lower water vapor permeability and enhanced mechanical properties. The antibacterial properties of the film against *Pseudomonas aeruginosa* (*P. aeruginosa)* and *S. aureus* strains have shown increments with an increase in curcumin concentration. However, the film demonstrated a lower antibacterial effect than the corresponding powder curcumin–TiO_2_ mixture. The pH-dependent color change of the film has been observed, implying the film can indicate the suitability of the product for consumption. Another study reported curcumin ZnO incorporated carboxymethyl cellulose-based functional film. 1 wt% curcumin and 1 w% ZnO incorporated film demonstrated optimal antibacterial and antioxidant properties without compromising the optical transparency and mechanical property of the film [95]. Issa M. et al. [96] studied the effect of cellulose binder on the ZnO-cast cotton fabric on the antimicrobial and wash fastness property of the composite fabric. They observed that the use of cellulose binder enhanced the antibacterial property of the fabric against *E. coli* and *S. aureus*. The incorporation of curcumin and Ag together with ZnO further promoted the antibacterial properties of the fabric.

A wound-dressing patch synthesized by casting curcumin–TiO_2_ incorporated chitosan (CS) solution on nonwoven polypropylene cloth has been reported by Marulasiddeshewawa et al. [75]. TiO_2_ incorporation into the patch led to a lower drug release rate compared to patches without TiO_2_. The in vitro antibacterial activity of the patch against *E. coli* and *S. aureus* revealed that a TiO_2_ containing patch has a better antimicrobial effect than CS and CS/curcumin. An in vivo wound healing analysis on MRSA-inoculated mice xenograft also has revealed the better performance of CS/TiO_2_/curcumin in terms of wound contraction, bacterial growth inhibition, re-epithelialization, and good collagen organization compared to only CS membrane or CS/curcumin.

A sodium alginate (SA)-based curcumin–metal oxide-incorporated wound dressing patch has also been reported. The highly hydrophilic nature of SA makes it suitable for wound exudate absorption and damp environment maintenance. A study reported an efficient wound healing capability of TiO_2_-curcumin incorporated polyvinyl alcohol/sodium alginate composite patch (PVA/SA/TiO_2_-curcumin) patch. The water absorption rate (2124–2267 mg^−2^day^−2^) and the hemolytic assay (4.8% at 180 min) of the patch were found to be within the optimum limits, 2000–2500 mg^−2^day^−2^ and less than 5% lysis, respectively. PVA/SA/TiO_2_-curcumin patch demonstrated biocompatibility towards NIH3T3 cell lines and antibacterial activities against *B. subtilis*, *S. aureus*, *E. coli*, and *P. aeruginosa*. They observed that TiO_2_ incorporation in the patch resulted in controlled drug release than PVA/SA–curcumin. In vivo wound healing analysis on incision wound-induced 18 Wistar albino rats revealed the better performance of PVA/SA/TiO_2_-curcumin as a wound dressing patch in terms of supporting complete re-epithelialization of the skin. The author attributed the antimicrobial performance to the combined effect of curcumin and TiO_2_ nanoparticles [97]. In another study, curcumin–TiO_2_ incorporated sodium alginate (SA) and polyvinyl alcohol (PVA)-based patch has been synthesized using a gel-casting method and its antimicrobial performance was studied in vitro. The synthesized patch has shown a different degree of inhibition of the different bacteria (i.e., at 100 µg concentration of TiO_2_, 12 mm, 12 mm, 10 mm, and 9 mm zone of inhibition against *B. subtilis*, *S. aureus*, *E. coli*, *Klebsiella pneumonia* (*K. pneumonia),* respectively). The antimicrobial effect of the patch has been better than that of the common antibiotic clotrimazole. However, they observed the antimicrobial effect of the patch to be lower than that of powdered TiO_2_ nanoparticles [98].

Physical adsorption of ZnO–curcumin nanocomposite on collagen skin wound dressing material has been reported. Antibacterial activity of the nanocomposite loaded collagen membrane against clinically isolated coagulase-negative *Staphylococci* (CoNS) revealed significant cell death compared to viable control. Treatment of CoNS with the nanocomposite membrane resulted in cell clustering and lack of cell integrity [99].

### 3.4. Curcumin-Metal Oxides Incorporated Hydrogels

Hydrogels are three-dimensional porous and physically or chemically crosses-linked networks of water-soluble polymers [100]. They can absorb a large amount of water and biological fluids [17]. Their porous structure and swelling property in aqueous media make them suitable for drug loading and delivery. George et al. [101] have observed the incorporation of ZnO NPs in dimethyl cellulose crosslinked chitosan hydrogel promoted the drug loading capacity of the gel. They obtained a 30% increment in loading efficiency by incorporating ZnO nanoparticles. The nanoparticle incorporation also has enhanced the mechanical properties of the gel. They attributed the higher loading to the electrostatic repulsion between zinc oxide NPs and consequent wider pore formations. A curcumin-loaded, ZnO incorporated hydrogel demonstrated less cytotoxicity towards fibroblast epidermal (L929) while at the same time causing the highest cytotoxicity against skin carcinoma (A431). They attributed the selective cytotoxicity of the nanocomposite to the synergistic effect of ZnO NPs and curcumin. Andarabi et al. [102] have also obtained better mechanical stability and stable elastic behavior by incorporating ceria and curcumin in gelatin–glucan-based hydrogel. The pure hydrogel demonstrated a ~252% swelling ratio, and nanoparticle incorporation had not reduced the swelling ratio significantly. The hydrogel has shown faster degradation (98% in 15 days), favoring cell proliferation and consequent wound healing. Release behavior of the hydrogel system (GCCe) was controlled and continuous throughout, 63% in 108 h. Hemolytic assay, cytotoxicity towards HaCat cell, and RRIS scavenging ability from H_2_O_2_ treated HaCat demonstrated that the hydrogel was biocompatible and had antioxidant property. Another study reported hybrid hydrogel from organic poly-aspartic using inorganic graphene and poly (acrylamide-co-acrylic acid) as a primary and secondary crosslinker, respectively. The incorporation of silver, CuO, and ZnO in the hydrogel increased its swelling behavior. The curcumin-loading efficiency of the hydrogel increased with an increase in Ag content. However, the opposite effect has been observed by increasing ZnO and CuO contents. The stability of curcumin in the hydrogel and corresponding controlled release has been possible by the incorporation of NPs in the hydrogel. Nanoparticles incorporated hydrogel demonstrated bacterial growth reduction. The antibacterial efficacy of the composite hydrogel is attributed to the synergistic effect of NPs, graphene, and curcumin [103].

### 3.5. Curcumin-Ameliorated Metal Oxide Toxicities

Metal oxides are used in many products that have direct contact with our body, such as cosmetics, toothpaste, food colorants, and food packaging. Some of these products may lead to the ingestion of metal oxide NPs. In vitro and in vivo studies have revealed that exposure to a large number of metal oxides, like ZnO, CuO and TiO_2_, for extended periods may cause damage in liver cells, cytotoxicity to bronchial epithelial, cytotoxicity reproductive cells, and genotoxicity.

A study demonstrated the protective effect of curcumin against ZnO NP induced liver cell damages. In vivo studies on ZnO NPs (20–3 nm particles at a dose of 50 mg/kg per day for 14 days) ingested mice revealed a high concentration of serum toxicity markers like ALT, AST and ALP, which were 2.7, 28, and 1.97 times higher than the control groups. However, curcumin and ZnO-treated mice demonstrated lower concertation of toxicity markers than ZnO-only-treated mice. MDA levels and SOD and GPx activities show no change in curcumin-treated mice, but ZnO intoxicated mice show two-fold MDA levels. Curcumin administration with ZnO reduces MDA level by 39%. SOD and GPx activities were also higher in the case of curcumin and ZnO samples compared to ZnO only treated mice. Histological analysis shows necrosis in the case of ZnO-treated mice; the co-administration of curcumin improves the structures of liver-reducing the level of necrosis. The positive effect of curcumin can be associated with its antioxidant property [104]. Amer et al. [105] studied the ameliorative effect of curcumin on ZnO NPs caused deterioration of the cerebral cortex. Mice exposed to ZnO NPs (IP-injected 5.6 mg/kg body weight, 3 times per week for 28 days) demonstrated a lower rate of body gain, significant increment in cerebellum Zn level, altered cerebellar histology, Purkinje-cell-density decrease, increase in apoptotic markers, overall cerebral cortex deterioration. Co-administration of curcumin with ZnO significantly prevents cerebral cortex damage and resulted similar histology and level of toxicity markers with control group. Another study evaluated the ameliorative effect of curcumin against TiO_2_ NPs caused reproductive problems. In vivo study revealed TiO_2_-nanoparticle-treated mice (50 mg/kg for 35 days) show a significant reduction in testicular weight, testosterone concentration, morphometric parameters, and sperm quality. Pretreatment of the mice with curcumin demonstrated effective attenuation of the events [106]. Elkhateeb et al. [107] analyzed the ameliorative effect of curcumin on CuO-induced renal toxicity. Treatment of mice with 250 mg/kg of body weight CuO for three months resulted in all signs of oxidative stress, inflammation, and histopathological alteration in kidney structure. Pretreatment of mice with curcumin improved most of the adverse effects caused by CuO treatment.

## 4. Concluding Remarks

Metal-oxide nanoparticles (MONs) are emerging as preferred nanocarriers in delivering several therapeutic drugs. They can offer additional functionalities besides carrying the payload, such as molecular probing, serving as contrasting agents in diagnosis, and providing additional therapeutic effects like photodynamic therapeutic effect and hyperthermia. Several studies have demonstrated the multifunctional benefit that metal oxides can offer in the biomedical fields. The use of metal oxides in drug delivery, specifically in the delivery of hydrophobic curcumin, can be referred to as limited compared to the overwhelming number of studies on the use of organic and other inorganic carriers. Thus, there is a need for a greater number of studies on designing metal oxides as an efficient carrier for curcumin delivery.

Several parameters determine the properties and the corresponding effectiveness of metal oxides in the biomedical fields. The particle size, particle size distribution, phases in the case of polymorphic metal oxides, morphology, surface area, surface characteristics, impurities, and chemistry of the MONs determine their effectiveness in biomedical applications. However, the majority of the research so far has given less emphasis on optimizing the characteristics of MONs to get the best performance in their biomedical applications. In most research, the emphasis has been given to the type of metal oxide used and on the surface modification of MONs with other organic and inorganic materials. Although playing with the material type and surface modification have proven to bring about better performance in the biomedical applications of MONs, an even wide possibility can be obtained by manipulating the physical and chemical characteristics of MONs.

Most of the research on the biomedical applications of MONs, specifically on the drug delivery of curcumin by MONs, is based on preclinical studies (more of in vitro and a little in vivo experiments). Thus, still, there need to be enough in vivo studies to clearly correlate the performance of MONs-based curcumin delivery with their characteristics in real biological environments, and subsequent advancement towards further clinical trials needs to be made.

Metal oxides have shown dose-dependent toxicities in several studies. In most studies, these toxicities are correlated to the concentration of the metal oxides only and do not give a comprehensive understanding unless other important parameters like phase morphology, and surface property, which determines toxicities of MONs, are considered. Thus, thorough studies that can give a complete picture of the range of metal oxides toxicities considering all the influential parameters needs to be done.

## Figures and Tables

**Table 1:** Direct combination of metal oxides and curcumin and their therapeutic performance.

Therapeutic Agent	Synthesis Approach	Study Models	Outcomes
Curcumin–TiO_2_ nanoparticles (CNTPs) [51]	Coprecipitation in the presence of curcumin	In vitro: THP1 and H9c2 In vivo: Sprague-Dawley rat model	↑ Stability curcumin in CTNPs form at pH 7.4 pH-dependent release at pH 6 > pH 7.4 In vitro: no distinct change in cells morphology, viability > 97% → nontoxicity of CTNPs In vivo: better biodistribution with more concentration in liver and kidney, half-life CTNPs > curcumin in all organs 24 h, high life of CN No change in SGPT, SGOT and LDH in CNTPs treated mice serum, no genotoxicity → in vivo nontoxicity of CTNPs
Curcumin-coated TiO_2_ nanotubes (TNTC) [52]	Drop-casting method	*E. coli*, *S. aureus* hMSCs	In vitro: ↓ wettability → reduced microbial adhesion, 43.6% *E. coli* and 38.5% *S. aureus* growth inhibition, no cytotoxic effect on hMSCs, ↓ ALP expression
Curcumin/TiO_2_ nanocomposites [53]	Adsorption from solution under sonication	HeLa	↑ Inhibition of HeLa
Zinc oxide-grafted curcumin nanocomposites (ZNP-Cs) [20],	Surface adsorption from solution	*S. aureus*, *S. epidermidis*, *B. cereus*, and *E. coli* HEK293	Modest curcumin-loading Antibacterial activities rod, RZNP–cur> spherical, SZNP–cur > long petal, LPZNP–cur > javelin, JZNP–cur Oblong particles showed higher activities than spherical ones *E. coli* less susceptible to antimicrobial activities, SZNP–cur show the most potent against *S. epidermidis*, RZNP–cur show the most potent against the other three Composites demonstrate better performance than bare curcumin or ZnO
Zinc oxide–curcumin core–shell (ZnO–cur) [55]	Precipitation under	*S. aureus*, *S. pneumoniae, E. coli*, and *S. dysenteriae*	↑ Bacterial inhibition effect than amoxicillin and pure curcumin
Copper oxide–curcumin core–shell [57]	Precipitation under ultrasonication	*E. coli*, *S. aureus*, *S. dysenteriae*, and *S. pneumoniae*	↑ Water dispersibility, better aqueous solubility of curcumin in core–shell form than in pure form, ↑ Antibacterial effect than nanocurcumin and amoxicillin ↓ Toxicity towards African green monkey kidney cell (Vero) than CuO
Curcumin–CuO physical mixture [58]	Grinding together curcumin and CuO powders	Streptozotocin (STZ) induced diabetic mice	↓ MDA level, ↑ GSH and SOD levels, insulin concentration, mRNA expression level of insulin gene (IR-A and IR2)
Curcumin-coated iron oxide (C–IO) [59]	Coprecipitation in the presence of curcumin	HUVEC	↓ Toxicity compared to uncoated IO or curcumin to HUVEC Show protective effect to HUVEC from PCB126 induced toxicity

Abbreviations: ↑ = increase; ↓ = decrease; → = implies; SGPT =** serum glutamate-pyruvate transaminase, SGOT = serum glutamate-oxaloacetate transaminase; LDH = lactic dehydrogenase; ALP = alkaline phosphatase; hMSCs = mesenchymal stem cells; ZnO = zinc oxide; TiO2 = titanium dioxide; CuO = copper oxide; IO = iron oxide; RZNP–cur = curcumin-loaded rod shape ZnO; SZNP–cur = curcumin-loaded spherical shape ZnO; LPZNP–cur = curcumin-loaded long petal shape ZnO; JZNP–cur = curcumin-loaded javelin shape ZnO; HEK293 = human embryonic kidney cells; HUVEC = human umbilical vein endothelial cells; PCB126= polychlorinated biphenyl 126.

**Table 2:** Organic and inorganic surface modification in curcumin–metal oxide nanoformulations and corresponding therapeutic performance.

Therapeutic Agent	Synthesis Approach	Study Models	Outcomes
Folic acid (FA)/polyethylene glycol (PEG)/TiO_2_ [61]	Emulsion evaporation solidification	In vitro: MCF-7, MDA-MB-231, H9c2 In vivo: MDA-MB-231 tumor-bearing BALB/c mice	In vitro: ↑ viability of H9c2 compared to only TiO_2_ treatment ↑ Cytotoxicity towards MCF-7 and MDA-MB-231 cells, cellular uptake by MCF-7 cells compared to untargeted NPs In vivo: ↑ drug concentration at the tumor site and better antitumor activity compared to untargeted NPs
PTX–cur-OAMNPPF127FA [62]	Fe_3_O_4_ by coprecipitation, surface functionalization by deposition from solution	MCF-7	Lower hemolytic assay 4.1% ↑ Cellular uptake and growth inhibition of MCF-7 for active FA and passive magnetic field targeting Superparamagnetic behavior retained through lower saturation magnetization compared to MNP only
5FU–CUR-C-MNP-HSA-FA [63]	MNPs by coprecipitation	MCF-7	↑ Colloidal stability, Superparamagnetic property maintained, but less saturation magnetization ↑ Cytotoxicity against MCF-7 ↑ Cellular uptake ↑Viability of MCF-7 cells, for FA targeted under magnetic field
Folic-acid-tagged aminated starch/ZnO-coated Fe_3_O_4_ [64]	Stirring together the nanocomposite and curcumin suspension overnight and genipin crosslinking	HepG2 and MCF7	higher colloidal stability with zeta potential value of 42.9 mV, pH-dependent release profile → higher release rate at acidic pH, biocompatibility with human lymphocytes ↑ Cellular uptake and anticancer effect on MCF-7& HepG2
Curcumin-incorporated TiO_2_-conjugated with MCP-1 antibody (CTNP-MCP-1) [65]	Suspending presynthesized CTNPs in MCP-1 antibody under stirring	Cholesterol-fed, atherosclerotic Sprague-Dawley rat	Normal ALP, GGT levels, RBC morphology, and aorta architecture for CTNP-MCP-1 treated rats → nontoxic ↑ Aortic concentration of CTNP-MCP-1 compared to CTNP → better targeting ↑ Half-life, distribution, and ↑ hydrolysis of curcumin in CTNPs → better stability MRI image contrast of CTNP-MCP-1 > CTNP due to targeting effect of MCP-1 antibody and ↑ paramagnetic property of TiO_2_ when combined with carbon compounds
FA-mPEG–PAMAM G3-CUR@SPIONs [12]	Coprecipitation for Fe_3_O_4_, dispersion of the constituents under sonication and mixing	KB and MCF-7	↑ KB cells (with higher FRs) lethality compared to MCF-7 (with lower FRS)
TiO_2/_polydopamine (pDA)/polycyclodextrin (pDC)-curcumin [66]	Hydrothermal synthesis of nanotube array on Ti substrates, followed by pDA and pCD coating and curcumin-loading	In vitro: MG63, In vivo: osteocarcinoma xenografted nude mice	Membrane shrinkage, absence of filopodia and lamellipodia, ↓ Cell density of MG63 Biocompatibility towards MC3T3-E1 cells ↓ Tumor growth on osteocarcinoma xenografted nude mice
DOX and curcumin-loaded, HAPA/β-CD/SPION [67]	Fe_3_O_4_ by coprecipitation HAPA coating by coprecipitation, loaded β-CD functionalization and drug loading by mixing NPs with components in a suspension	In vitro: MCF-7 In vivo: tumor-bearing BALB/c mice	↓ Amount of protein corona adsorbed on the nanocomposite ↑ Cellular uptake by MCF-7 cells↑ Hemocompatibility with only 0.1% hemolysis ↑ Toxicity against MCF-7 cells ↓ percent relative tumor volume (% RTV) in the case nanocomposite with magnetic field treatment
MNP-CUR [68]	Fe_3_O_4_ by coprecipitation in the presence of β-CD and F68, curcumin-loading by diffusion method	MDA-MB-231	less aggregated nanocomposite Slow and sustained release of curcumin ↑ Drug uptake by the MDA-MB-231 cells ↑ Prevention of serum protein adsorption and colony formation ↓ Viability of MDA-MB-231 cells ↓ T2-weighted signal in MRI imaging analysis
Curcumin-loaded PEG/β-CD/ZnO [69]	Wet coprecipitation method	*S. aureus*, MCF-7	↑ Drug encapsulation efficiency, pH-dependent sustained drug release ↑ Antibacterial and anticancer properties compared to free curcumin ↑ Photoluminescence property compared to composite without β-CD
Curcumin-loaded β-cyclodextrin and pluronic F127 stabilized iron oxide (MNP–cur) [70]	Fe_3_O_4_ by Coprecipitation in the presence of β-cyclodextrin pluronic F127, curcumin-loading precipitation forms a suspension	In vitro: HPNote images cannot be edited for English. Please verify all text carefully.-II, Panc-1 A2780PC, MDA-MB-231, PC-3 In vivo: Note images cannot be edited for English. Please verify all text carefully.AF-II xenograft mice	↑ Water dispersibility efficient drug encapsulation and sustained release ↑ MRI contrasting during MRI scanning of A2780PC ↑ Inhibition of A2780PC, MDA-MB-231 ↑ Drug uptake by HPAF-II and Panc-1 ↑Proliferation and colony formation of HPAF-II and Panc-1 ↓ Tumor growth of HPAF-II xenograft mice ↑ Serum bioavailability of curcumin (2.5× that of free curcumin)
Curcumin-loaded chitosan (CS)–Fe_3_O_4_ [73]	Fe_3_O_4_ by reverse microemulsion, chitosan coating by stirring together CS in acidic solution and Fe_3_O_4_ suspension in the presence of CTAB	A549	controlled drug release profile, intact paramagnetic property, ↑ Cytotoxicity against A549 cells compared to free curcumin
Cur–O–CMCS–ZnO [74]	ZnO NPs using the coprecipitation method, O-CMCS coating using ex situ grafting, curcumin-loading, precipitation from solution	MA104, L929	↑ Water solubility of curcumin in the composite form ↑ Accumulation and cytotoxicity towards MA104 compared to L929
Curcumin-loaded, phendione-modified chitosan-coated CuO [76]	CuO by chemical reduction method, CS coating and curcumin-loading by precipitation from solutions	MCF-7, M19-MEL, HeLa	↑ Drug loading efficiency (96.3%), sustained drug release ↑ Anticancer effect (> 2×) M19-MEL, MCF-7, HeLa compared to free curcumin
Curcumin-loaded, chitin–glucan-coated ZnO NPs(Cur–ChGC@ZNONPs) [78]	ChGC@ZnONPs by coprecipitation in the presence of ChGC, curcumin-loading precipitation from solution	*E. coli* and *B. subtilis*	Better inhibition effect against *E. coli* and *B. subtilis* ↑ Radical scavenging activities → increased antioxidant properties
Curcumin-loaded PMMA–PEG/ZnO [94]	ZnO by coprecipitation, PMMA–PEG by double emulsion, curcumin-loading and copolymer coating precipitation from suspension.	AGS	↑ Drug loading percentage, 47% and loading efficiency, 92%, pronounced DNA fragmentation ↑ Anticancer effect compared to ZnO or curcumin
Curcumin-loaded, PEG-functionalized, gold nanoparticle decorated, Fe_3_O_4_–silica core–shell [81]	Coprecipitation for Fe_3_O_4_, surface modification and drug loading precipitation from solution	HL-60	Stable composite assembly with a zeta potential of -24.5 mV ↑ T2 value during MRI imaging, ↓ Viability of HL60, 53.4% in 42 h
Curcumin and PAC loaded PEG-modified CeO_2_@SiO_2_ (CeO_2_@SiO_2_–PEG–PAC/Cur) [80]	CeO_2_@SiO_2_ by chemical precipitation method, PEG by nanoprecipitation, drugs loading by precipitation from respective solutions	L02, HepG2, HeLa, PC-12	↓ Hydrodynamic size of the composite from 421 to 359 nm↓ Cytotoxicity towards L02, Neuroprotective effect against Aβ_1-42_ mediated PC-12 ↑ Antiproliferative effect against HepG2 and HeLa
cCur/ZnONPs [85].	cCur/ZnO by precipitation from solution	*Streptococcus mutans (S. mutans)*, *Streptococcus sobrinus (S. sobrinus)*, *Lactobacillus acidophilus* (*L. acidophilus)*	Antibacterial growth inhibition for 90 days, ↓ bacterial growth rate after 90 days shear bond strength in acceptable range (6–8 Mpa)
curcumin-loaded, oleic acid, and citric acid-functionalized Fe_3_O_4_ [84]	Fe_3_O_4_ by coprecipitation, in the presence of citric acid and oleic acid, curcumin-loading by precipitation from solution	MDA-MB-231	↓ Viability of MDA-MB-231 compared to free curcumin treatment superparamagnetic property with saturation magnetization 60–80 emu/g ↑ T2 relaxation time→ MRI contrasting potential
Curcumin–ZnO incorporated carboxymethyl cellulose (Cur/ZnO/CMC) [86]	ZnO/CMC by coprecipitation in the presence of CMC, precipitation from solution	L929, MA104	↑ Aqueous solubility, loading efficiency of 44%, controlled release profile ↓ Cytotoxic to L929 cells (80% viability) ↑ Cytotoxicity MA104 cells (20%, viability)
Curcumin-loaded carboxyl-terminated ZnO NPs [88]	Coprecipitation in the presence of 3-mercaptopropionic acid (MPA), curcumin-loading precipitation from solution	MDA-MB-23	↑ In aqueous and ↓ In IC_50_ against MDA-MB-23 compared to free curcumin
bovine serum albumin-coated Fe_3_O_4_–curcumin (F@BSA–curcumin) [89]	Desolvation and chemical coprecipitation process	HFF2, MCF-7	pH-dependent drug release biocompatible to HFF2 cells ↑ Cytotoxic to MCF-7
Curcumin-loaded, carbon dots-coated rutile TiO_2_ [91]	TiO_2_ by coprecipitation, carbon dot coating and Curcumin-loading precipitation from solution	HaCaT, MCF-7, McCoy	↑ Curcumin-loading percentage (89%) ↑ Inhibition of HaCaT comparable to that of the positive control 5-fluorouracil apoptosis on MCF-7, biocompatible to McCoy

Abbreviations: ↑ = increase; ↓ = decrease; → = implies; >= greater than; MCF-7 and MDA-MB-231 = breast cancer cell lines; H9c2 = cardio myoblast cell line; PTX–cur-OAMNPPF127FA = paclitaxel and curcumin-loaded oleic acid-functionalized, pluronic F127-coated, folic acid targeted magnetic nanoparticles; Fe_3_O_4_ = superparamagnetic iron oxide; 5FU–CUR-C-MNP-HSA-FA = 5 fluorouracil and curcumin-loaded citric acid and human serum albumin-functionalized, folic acid targeted magnetic nanoparticles; HepG2 = human liver cancer cell; ALP = alkaline phosphatase; GGT = gamma glutamyl transferase; RBC = red blood cell; MG63 = postoperative tumor cells; MC3T3-E1 = mouse embryonic osteoblasts; HAPA = hydroxy apatite; β-CD = β-cyclodextrin; DOX = doxorubicin; MRI = magnetic resonance imaging; PEG = polyethylene glycol; A2780PC = ovarian cancer cell; HPAF-II and Panc-1 = human pancreatic cancer cells; A549 = adenocarcinoma human alveolar basal epithelial cells; CTAB = cetrimonium bromide; ZnO = zinc oxide; O-CMCS = O-carboxylchitosan; MA104 = cancer cells; L929 = murine fibroblast; M19-MEL = Cellosaurus cell line; CuO = copper oxide; AGS = gastric cancer cells; PMMA = poly(methyl methacrylate); HL-60 = leukemia; CeO_2_ = cerium oxide, SiO_2_ = silicon dioxide; PAC = proanthocyanidin; L02 = normal human liver cell; PC-12 = rat adrenal pheochromocytoma cell lines; CMC = carboxy methyl cellulose; IC_50_ = concentration that cause 50% cell density reduction; HaCaT = keratinocyte skin cells; McCoy = mouse fibroblast cells.

**Table 3:** Curcumin–metal oxide incorporated films, patches, and hydrogels and their corresponding therapeutic performances.

Therapeutic Agent	Synthesis Approach	Study Models	Outcomes
CMC/curcumin/ZnO film [77]	Solution-casting method	L. monocytogenes and *E. coli* DPPH• and ABTS•+	↓ Visible-light transparency, UV transmittance blocked, ↑ Mechanical strength and stiffness, but ↓ flexibility ↓ WVP, optimal antibacterial and antioxidant properties
SSPS/TiO_2_ nanoparticles/curcumin film [95]	Solution-casting method	*P. aeruginosa* and *S. aureus*	↓ WVP, ↑ mechanical, antimicrobial effect, pH-dependent color change
Nonwoven polypropylene/chitosan/curcumin TiO_2_ (MCUT) [75]	Suspension-casting	In vitro: *E. coli* and *S. aureus* In vivo: MRSA-inoculated Sprague–Dawley rats	Moderated water uptake, delayed drug release pattern In vitro ↑ antibacterial effect In vivo: better wound contraction, bacterial growth inhibition, re-epithelialization, and good collagen organization
PVA/SA/TiO_2_–cur [97]	Slurry-casting	In vitro: *B. subtilis*, *K. pneumonia*, *S. aureus*, *E. coli,* *Candida albicans (C. albicans)*, *Aspergillus niger* (*A. niger)*	Anti-fungal effect > clotrimazole, but < fluconazole antibiotics Effective antibacterial effect at 100 µg concentration for all bacteria than streptomycin antibiotics
Sodium alginate SA/PVA/TiO_2_/curcumin patch [98]	Gel-casting method	In vitro: *B. subtilis*, *S. aureus, P. aeruginosa* In vivo: incision-wound-induced 18 Wistar albino rats	Good swelling rate → capable of absorbing exudate, water absorption rate and hemolytic assay within optimal limits, antibacterial effect against both Gram-positive and Gram-negative bacteria Nontoxic towards NIH3T3 cells In vivo: ↓ necrosis, complete re-epithelialization, uniform collagen and fibrous tissues
ZnO–curcumin incorporated collagen wound dressing [99]	Dip-coating of commercial collagen skin-wound dressing material in curcumin/ZnO suspension	CoNS	↑ CoNS cell death compared to viable control, CoNS cell clustering and lack of cell integrity
ZnO NPs in dimethyl cellulose-crosslinked chitosan hydrogel [101]	Solution-based preparation in the presence of ZnO NPs then soaking dried gel in curcumin solution	L929, A431	↑ Drug loading capacity (30% ↑ by incorporating ZnO) enhanced the mechanical properties ↑ Cytotoxicity towards L929↑ Cytotoxicity against A431
Ceria and curcumin in gelatin–glucan-based hydrogel [102]	Dispersion of curcumin and CeO_2_ in the hydrogel by physical interactions	HaCat	↑ Mechanical stability, stable elastic behavior, no significant ↓ swelling ratio by incorporating ceria in hydrogel Faster degradation, controlled drug release behavior Biocompatible with HaCat, Show antioxidant property against H_2_O_2_ treated HaCat

Abbreviations: ↑ = increase; ↓ = decrease; → = implies; CUR = curcumin; > = greater than; < = less than; CMC = carboxymethyl cellulose; ZnO = zinc oxide; DPPH• = 2,2-diphenyl-1-picrylhydrazyl; ABTS = 2,2′-azino-bis(3-ethylbenzothiazoline-6-sulfonic acid); UV = ultraviolet; SSPS = soluble soybean polysaccharide; WVP = water vapor permeability; TiO_2_ = titanium dioxide; MRSA = methicillin-resistant *Staphylococcus aureus*; PVA = polyvinyl alcohol; SA = sodium alginate; NIH3T3 = murine fibroblast cell, CoNS = coagulase negative *Staphylococci*; L929 = murine fibroblast; A431 = human skin carcinoma; HaCat = human keratinocyte; H_2_O_2_ = hydrogen peroxide.

## Data Availability

No new data were created or analyzed in this study. Data sharing is not applicable to this article.
